# Soil degradation influences soil bacterial and fungal community diversity in overgrazed alpine meadows of the Qinghai-Tibet Plateau

**DOI:** 10.1038/s41598-021-91182-7

**Published:** 2021-06-02

**Authors:** Lin Dong, Jingjing Li, Juan Sun, Chao Yang

**Affiliations:** Grassland Agri-Husbandry Research Center, College of Grassland Science, Qingdao Agricultural University, Qingdao, 266109 China

**Keywords:** Grassland ecology, Microbial ecology

## Abstract

Over half of the alpine meadows in the Qinghai-Tibet Plateau (QTP) are degraded due to human activities. Soil degradation from overgrazing is the most direct cause of grassland degradation. It is thus important to synthesize the effects of multiple soil degradation indicators on the belowground biomass of plants and soil microorganisms in the degraded QTP. We studied the diversities and structures of soil bacterial and fungal communities using soil bacterial 16S rRNA and the fungal ITS gene under four degradation gradients, D1: lightly degraded, D2: moderately degraded, D3: highly degraded, and a non-degraded control site (CK). The bacterial Shannon diversity in D3 was significantly lower than that in D1 (*p* < 0.001), and the bacterial richness index in D3 was significantly lower than that in D1 (*p* < 0.001). There was no difference in soil fungal diversity among the different degradation levels; however, soil fungal richness decreased significantly from CK to D3. The phyla *Actinobacteria*, *Acidobacteria* and the genus *Mortierella* were differed significantly under the four degradation gradients. Plant litter mass and root C/N ratio were important factors associated with bacterial and fungal diversity and richness. These results indicated that alpine meadow degradation can lead to variations in both microbial diversity and the potential functioning of micro-organisms in the QTP.

## Introduction

Grassland degradation can produce highly negative environmental impacts, such as desertification, dust storms, and soil erosion^1^. Alpine meadows, which account for 38% of the grassland area in the Qinghai-Tibet Plateau (QTP), are a principal component of the natural ecosystem^2^. However, over half of alpine meadows are degraded, largely due to human activities^3^, such as overgrazing^4,5^. Overgrazing has reduced above- and below-ground biomass^6,7^ and decreased diversity and structure in the plant community^8,9^. It has also reported that overgrazing has resulted in severe soil degradation due to 3.02 Pg of carbon being lost over the last 30 years in the QTP^10^.

Soil degradation caused by overgrazing is the most direct cause of grassland degradation^11^. Soil properties that have been proposed as indicators of soil quality include organic carbon, pH, water storage, bulk density, electrical conductivity, and nutrient availability^12,13^. More than one indicator is typically required for assessing the effects of tillage management systems^14^ as well as grassland ecosystems. Therefore, it is important to synthesize the effects of multiple soil degradation indicators on belowground plant biomass and soil micro-organisms in the degraded QTP.

The changes in soil nutrient availability and plant diversity caused by alpine meadow degradation can alter the microbial community and its diversity^15^. Soil water availability^16^, pH^17,18^, bulk density^19^, and plant composition and biomass^20^ are key factors responsible for changes in microbial communities. Therefore, the changes in soil physicochemical properties and plant attributes during the process of grassland degradation inevitably change the diversity and composition of the soil microbial community^6,21^. A previous study confirmed that *Actinobacteria*, *Proteobacteria*, and *Acidobacteria* were well adapted to the soil conditions in the degraded alpine steppes at Qinghai-Tibetan Plateau^15^. Li, et al.^6^ found no significant difference in bacterial species composition in moderately degraded alpine meadows; however, a shift from *Sordariomycetes* to *Dothideomycetes* within *Ascomycota* was found with increasing degradation level. The microbial alpha diversity might be higher in severely degraded alpine meadows^6^, but many studies have found no difference in microbial alpha diversity among different degraded steppes^15^. Given the inconsistent response of soil micro-organisms to grassland degradation^22^, understanding how both bacterial and fungal community composition and diversity respond to degradation, and clarifying the key soil factors related to degradation, could provide a basis for the health evaluation and management of alpine meadows in the QTP^6^. There are few studies on the diversity of bacterial and fungal communities in degraded alpine meadows in the QTP, and it is unclear if soil degradation is also related to the composition and structure of soil fungi.

Long-term overgrazing has produced different levels of soil degradation in the alpine meadow of northeastern Qinghai Province, China. In this study, we used high-throughput sequencing of soil bacterial 16S rRNA and the ITS gene region of fungi to study the diversity and structure of soil bacterial and fungal communities under four different degradation conditions. To better understand the potential functional contributions of the observed soil bacteria in the degraded alpine meadow, we targeted human disease functional classes by calculating the metabolic pathways of the 16S rRNA gene sequences in the KEGG (Kyoto Encyclopedia of Genes and Genomes) database^23^. We hypothesized that (1) long-term overgrazing caused shifts in both plant and soil properties, and (2) soil degradation significantly altered bacterial and fungal diversity. The goals of this study were to (1) identify the plant root and soil properties under different degradation conditions, (2) evaluate the response of soil bacterial and fungal communities to a degraded meadow, and (3) assess the relationships between soil degradation, plant root properties, and soil microbial diversities.

## Results

### Plant root property responses to grassland degradation

The plant RC and RC/N ratios significantly increased as the degradation level increased (*p* < 0.05, Table 1), while litter mass, plant RB, and RN significantly decreased (Table 1; *p* < 0.05). Additionally, the soil pH, EC, and BD values significantly increased as the degradation level increased (*p* < 0.05, Table S1), while the contents of SWC, TC, TN, and the C/N ratio significantly decreased (*p* < 0.05).

**Table 1:** One-way ANOVA of the soil properties of non-degraded (CK), lightly degraded (D1), moderately degraded (D2), and highly degraded (D3) site.

	Litter mass (g m^−2^)	Root biomass (g m^−1^)	Root C (g kg^−1^)	Root N (g kg^−1^)	Root C/N ratios	Soil degradation
CK	70.5 ± 0.6	522.6 ± 3.7	412.7 ± 1.0	17.2 ± 0.3	24.0 ± 0.4	− 0.85 ± 0.1
D1	63.3 ± 1.7	330.9 ± 8.3	416.9 ± 0.8	16.2 ± 0.2	25.7 ± 0.3	− 0.28 ± 0.2
D2	55.3 ± 2.3	354.2 ± 9.4	423.6 ± 0.9	14.6 ± 0.2	29.1 ± 0.3	− 0.29 ± 0.3
D3	33.5 ± 1.0	316.3 ± 5.6	427.8 ± 1.1	12.3 ± 0.2	34.9 ± 0.5	1.42 ± 0.03
One-way ANOVA						
F-value	106.5	179.9	48.38	95.89	137.6	26.25
* p*-value	< 0.001	< 0.001	< 0.001	< 0.001	< 0.001	< 0.001

Values are the mean ± standard error.

Significant relationships at *p* < 0.001 were calculated using Tukey’s pairwise tests.

**Figure 1:**
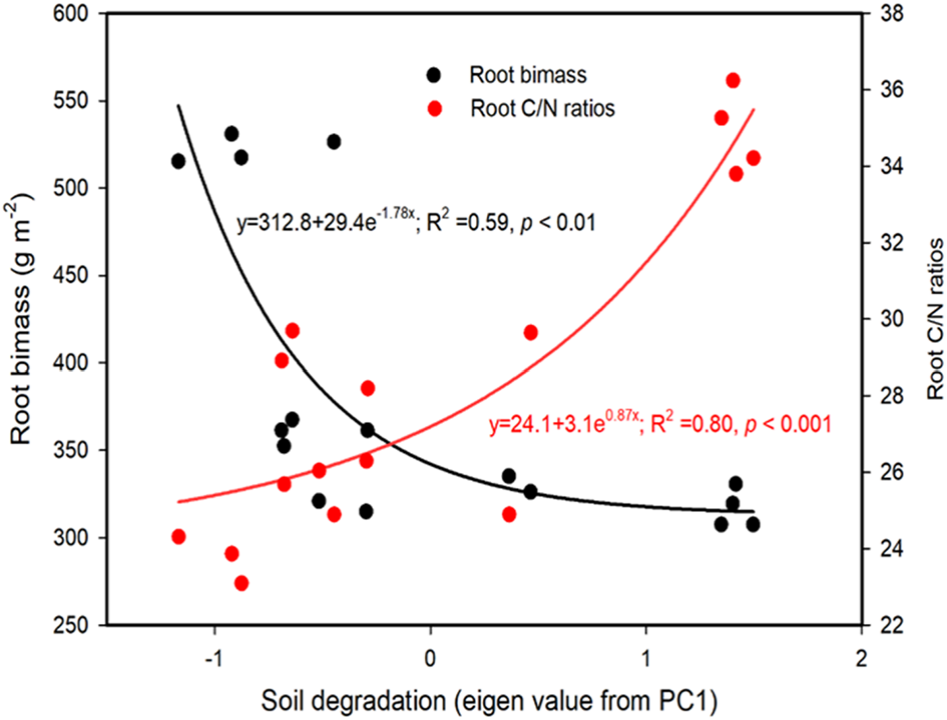
The correlation between soil degradation and plant root biomass and root C/N ratios.

In particular, the degree of soil degradation, obtained from PCA axis 1, also significantly increased as the degradation level increased (*p* < 0.05, Table 1). The non-linear regression revealed a negative correlation between soil degradation and plant root biomass (R^2^ = 0.59, *p* < 0.01); however, the non-linear regression showed a positive correlation between soil degradation and root C/N ratios (R^2^ = 0.80, *p* < 0.001) (Fig. 1).

### Soil microbial property responses to grassland degradation

**Figure 2:**
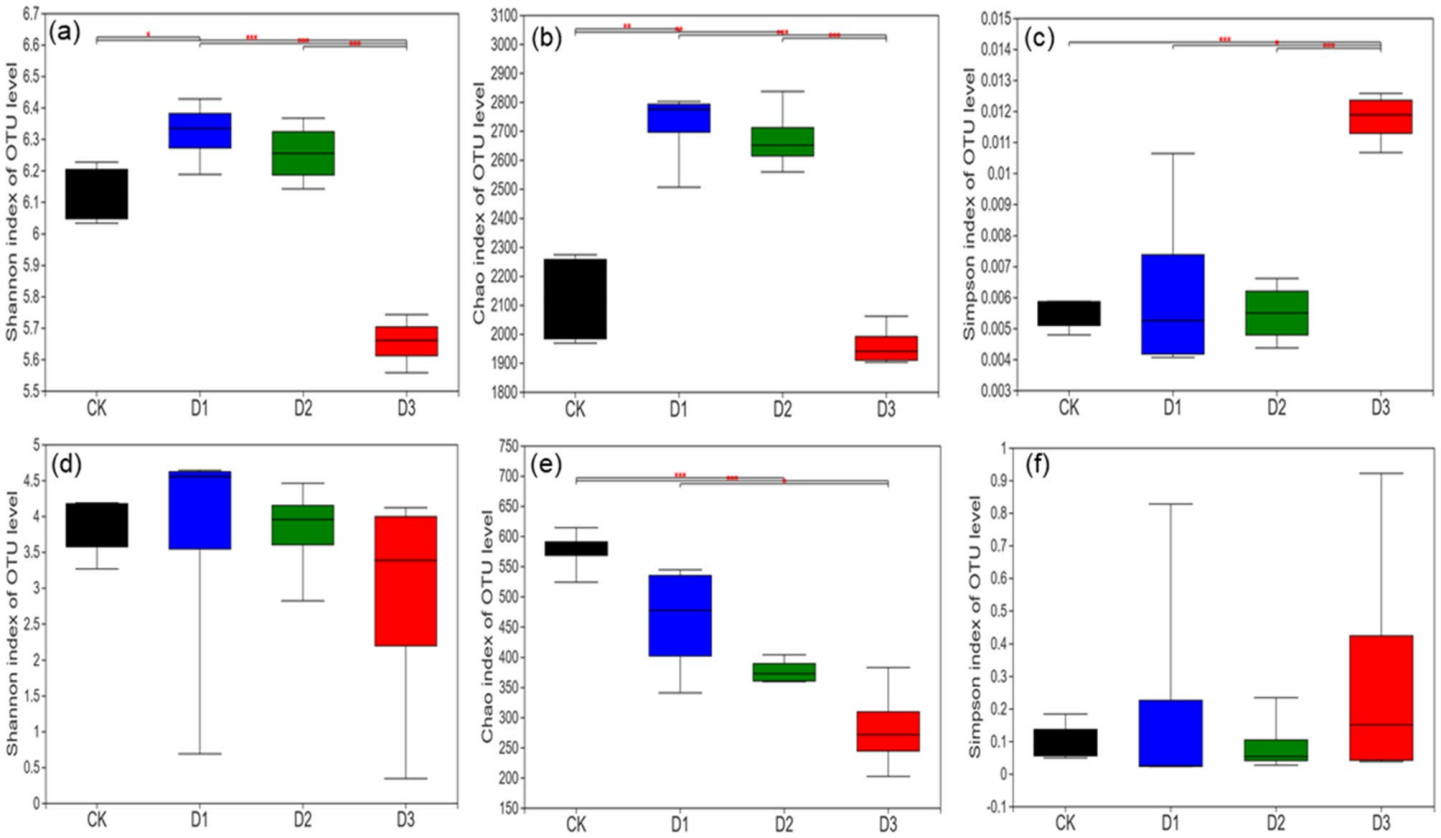
Bacterial and fungal diversity indices (**a**, **d** Shannon index; **b**, **e** Chao richness index; and **c**, **f**Simpson) calculated from 16S rRNA and ITS gene sequence data at the OTU level. *p < 0.05, **p < 0.01, and ***p < 0.001 based on Tukey’s honestly significant difference (HSD) tests.

The bacterial Shannon diversity index in D3 was significantly lower than that in the CK (Fig. 2a; *p* < 0.001), and the bacterial Chao richness index in D3 was significantly lower than that in D1 (Fig. 2b; *p* < 0.001); however, the bacterial Simpson index in D3 was significantly higher than that in CK, D1, and D2 (Fig. 2c; *p* < 0.001). There was no difference in the soil fungal Shannon and Simpson indices among the four different degradation levels (Fig. 2d, f); however, the soil fungal Chao richness index decreased significantly from CK to D3 (Fig. 2e).

**Figure 3:**
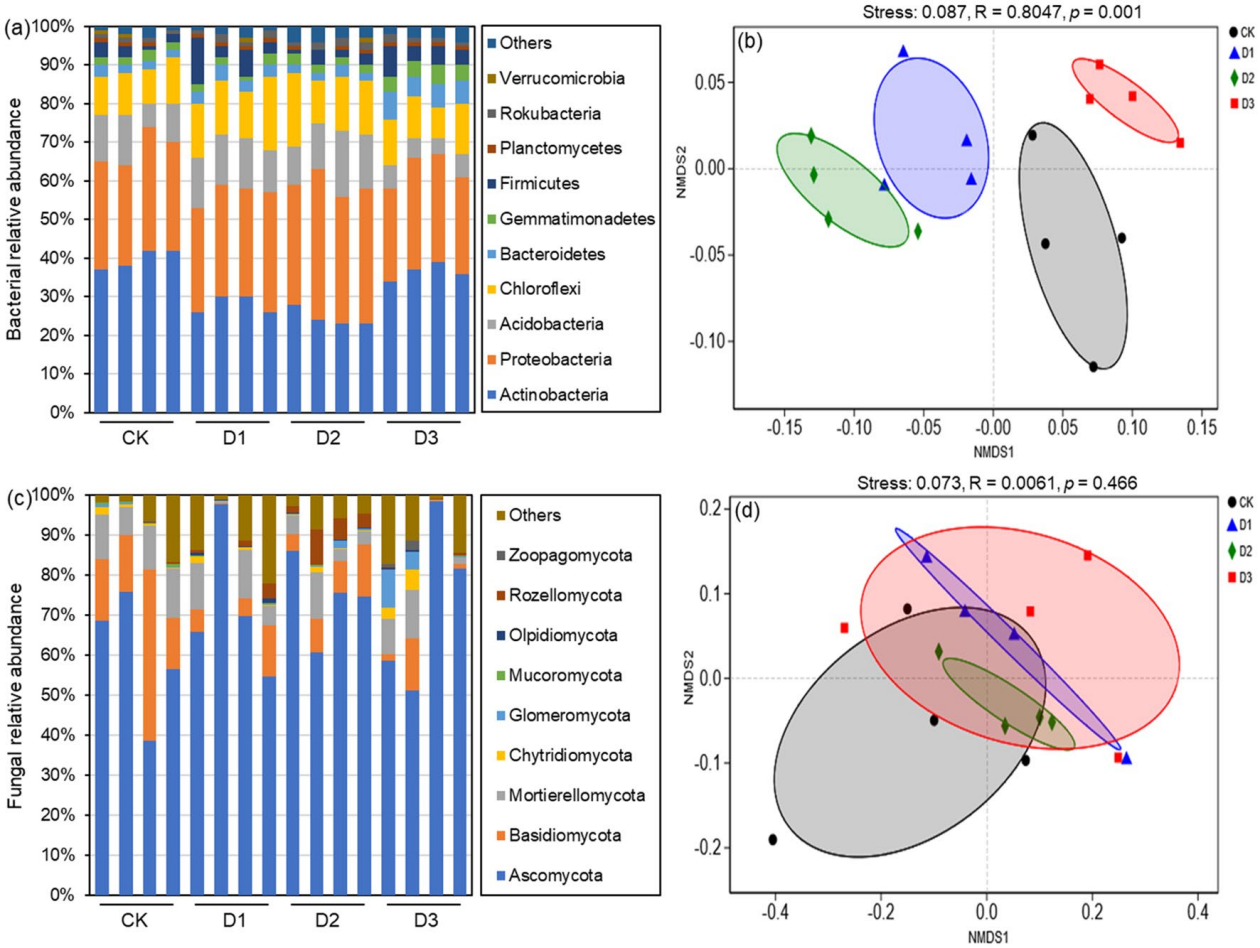
Relative abundances of the soil bacterial (**a**) and fungal (**c**) phyla in non-degraded (CK), lightly degraded (D1), moderately degraded (D2), and highly degraded (D3) alpine meadows, and nonmetric multidimensional scaling (NMDS) ordinations based on the mean abundance values of the soil bacterial (**b**) and fungal (**d**) phyla at the four different degradation levels.

*Actinobacteria*, *Proteobacteria*, *Acidobacteria*, and *Chloroflexi* were the main microflora at all four degradation levels, accounting for about 80% of the total bacterial abundance (Fig. 3a). At the genus level, *o_Actinomarinales* and *f_Geminicoccaceae* were the main microflora in D3 (Fig. S1a). The NMDS and PERMANOVA tests confirmed that the communities in D1, D2, and D3 were significantly different from those in the CK (R^2^ = 0.8047, *p* = 0.001; Fig. 3b). *Ascomycota* was the main microflora at all four degradation levels, accounting for about 60% of the total fungal abundance (Fig. 3c), and the genera *Mortierella*, *Pseudeurotium*, and *o_Tremellales* were the main microflora in the CK (Fig. S1b). The PERMANOVA tests showed that the communities in D1, D2, and D3 did not differ from those in CK (R^2^ = 0.0061, *p* = 0.466; Fig. 3d).

### Relationships between the shifts in soil properties, and microbial community and diversity

**Figure 4:**
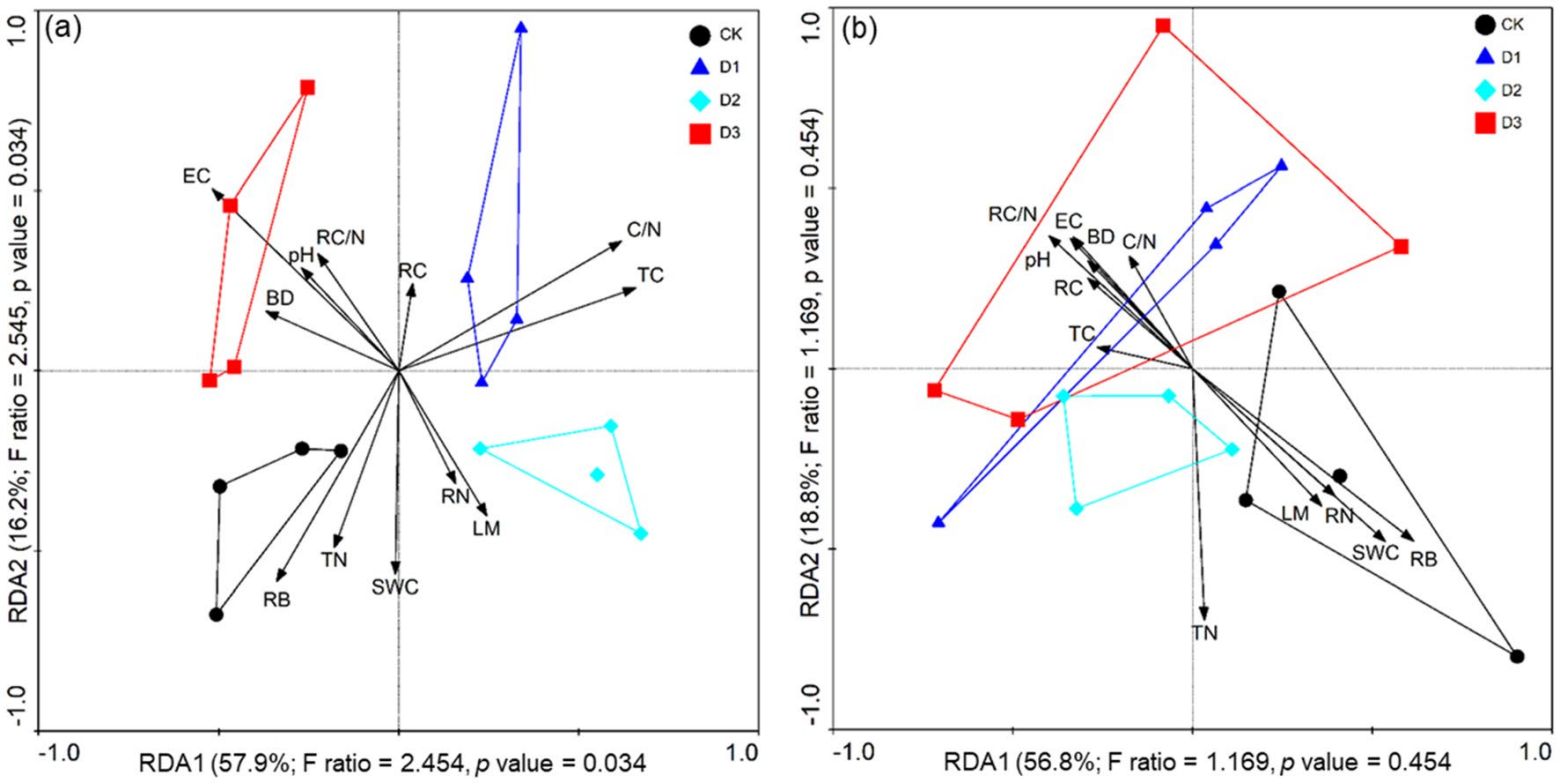
Redundancy analysis (RDA) showing the impact of plant litter mass, root properties, and soil physiochemical properties (pH, EC, BD, SWC, salt content, and C/N) on bacterial (**a**) and fungal (**b**) community structure. The significance of the effect of each property was tested using Monte Carlo permutation tests (permutations = 499).

**Figure 5:**
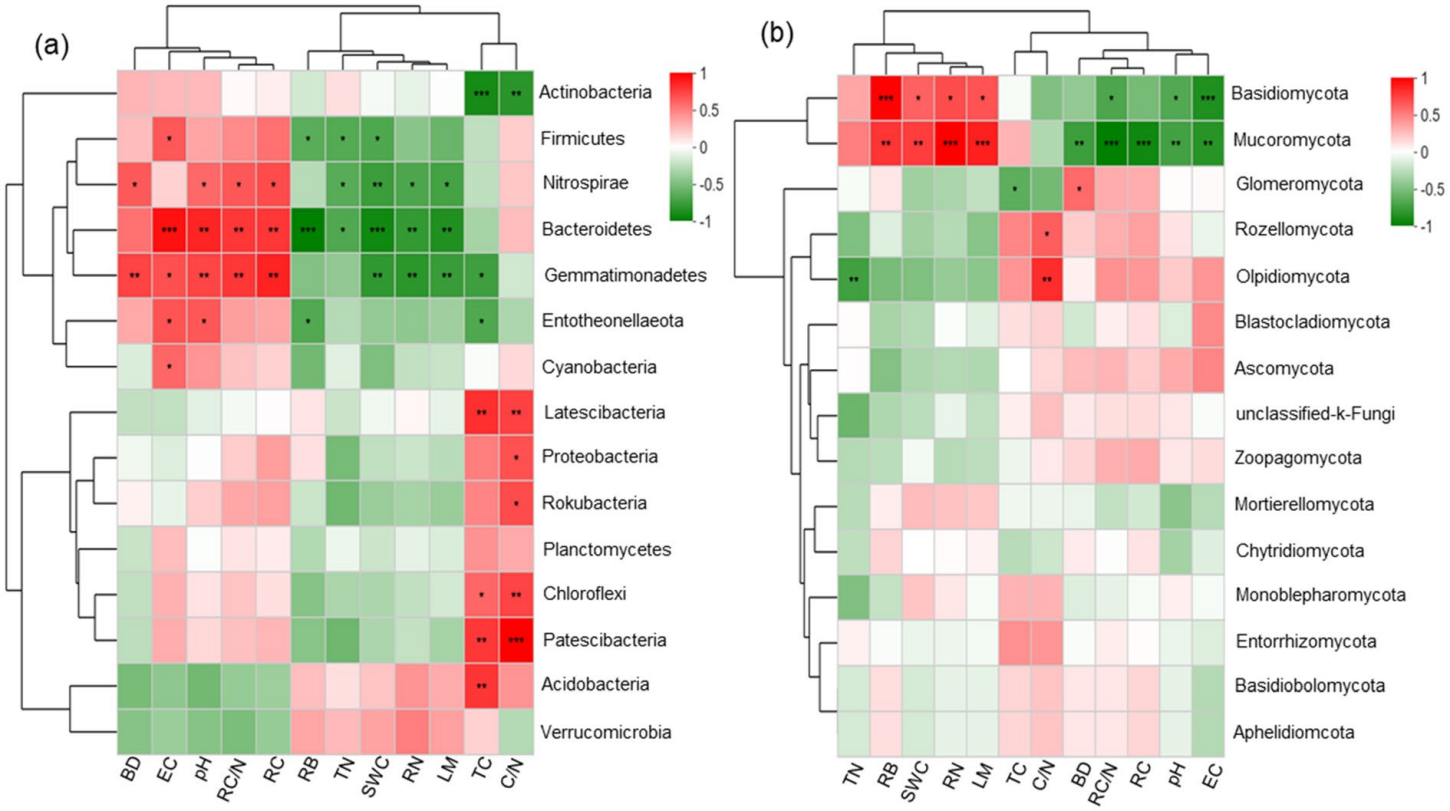
Spearman’s heatmap analyses between the relative abundances of specific bacterial (**a**) and fungal (**b**) genera and soil property parameters. EC (electrical conductivity), BD (bulk density), SWC (soil water content), TC (total carbon), TN (total nitrogen), C/N (soil total carbon/nitrogen), RB (root biomass), RC (root carbon), RN (root nitrogen), LM (litter mass), and RC/N (root carbon/nitrogen). *, **, and *** indicate the significance along the paths at the level of *p* < 0.05, *p* < 0.01 and *p* < 0.001, respectively.

In the RDA biplots, a combination of soil variables explained 74.1% and 75.6% of the bacterial and fungal community variance, respectively (Fig. 4). According to the Monte Carlo permutation test, plant root C, soil pH, EC, and soil TC significantly influenced the bacterial communities (Table S2), while only root biomass and soil TN significantly influenced the fungal communities (Table S2). The Spearman’s correlation heatmap showed that the abundance of *Bacteroidetes* was significantly positively correlated with soil EC (*p* < 0.001), pH (*p* < 0.01), root C/N (*p* < 0.01), and root C (*p* < 0.01), but negatively correlated with RB (*p* < 0.001), SWC (*p* < 0.001), RN (*p* < 0.01), and LM (*p* < 0.01) (Fig. 5a). The abundance of *Mucoromycota* was significantly positively correlated with soil RB (*p* < 0.001), SWC (*p* < 0.001), RN (*p* < 0.001), and LM (*p* < 0.001), but negatively correlated with root C/N (*p* < 0.001), root C (*p* < 0.001), pH (*p* < 0.01), and EC (*p* < 0.01) (Fig. 5b).

**Figure 6:**
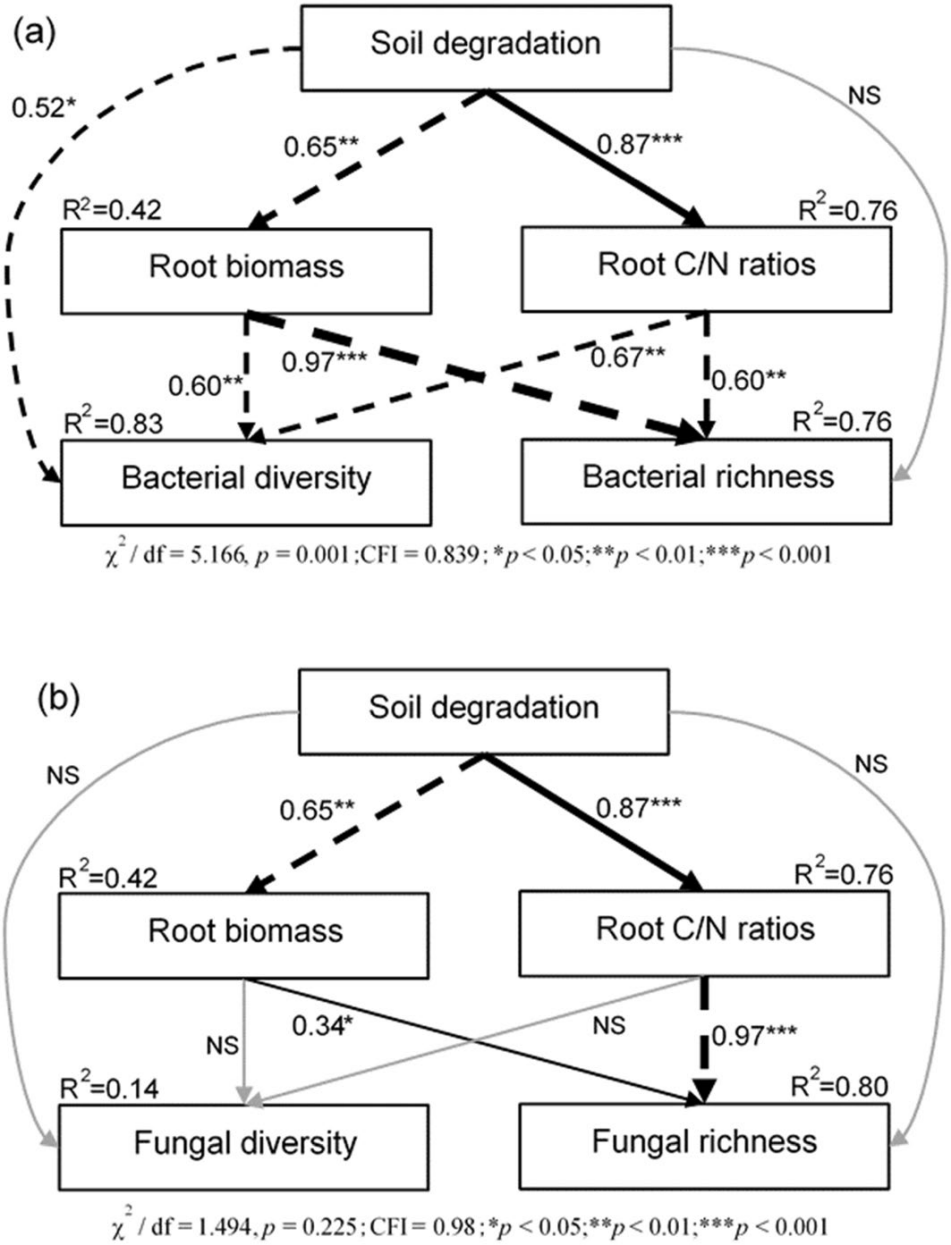
Structural equation model (SEM) based on the effects of soil degradation on soil microbial diversity and richness (**a**: bacteria, **b**: fungi). Continuous and dashed arrows indicate positive and negative relationships, respectively. Black and gray arrows represent significant and insignificant correlations, respectively. The width of the arrows is proportional to the strength of the path coefficients. R^2^ indicates the proportion of the variance explained and appears above every response variable in the model. The *, **, and ***indicate the significance along the paths at the level of *p* < 0.05, *p* < 0.01 and *p* < 0.001, respectively.

The SEM explained 83% and 76% of the variance in soil bacterial diversity and richness, respectively (Fig. 6a), and explained 14% and 80% of the variance in soil fungal diversity and richness, respectively (Fig. 6b). Soil degradation was significantly negatively correlated with plant root biomass (R^2^ = 0.65, *p* < 0.01), but positively correlated with root C/N ratios (R^2^ = 0.87, *p* < 0.001). Although soil degradation decreased the bacterial diversity, there was no significant correlation between soil degradation and soil fungal diversity. Additionally, plant root biomass was significantly negatively correlated with both soil bacterial diversity (R^2^ = 0.60, *p* < 0.01) and soil bacterial richness (R^2^ = 0.97, *p* < 0.001), and plant root C/N ratios were significantly negatively correlated with both soil bacterial diversity (R^2^ = 0.67, *p* < 0.01) and soil bacterial richness (R^2^ = 0.60, *p* < 0.01). However, plant root biomass (R^2^ = 0.34, *p* < 0.05) and root C/N ratios (R^2^ = 0.97, *p* < 0.001) were significantly positively and negatively correlated with soil fungal richness, respectively.

### Relationships between soil dergradation and metabolic pathways related to human diseases

**Figure 7:**
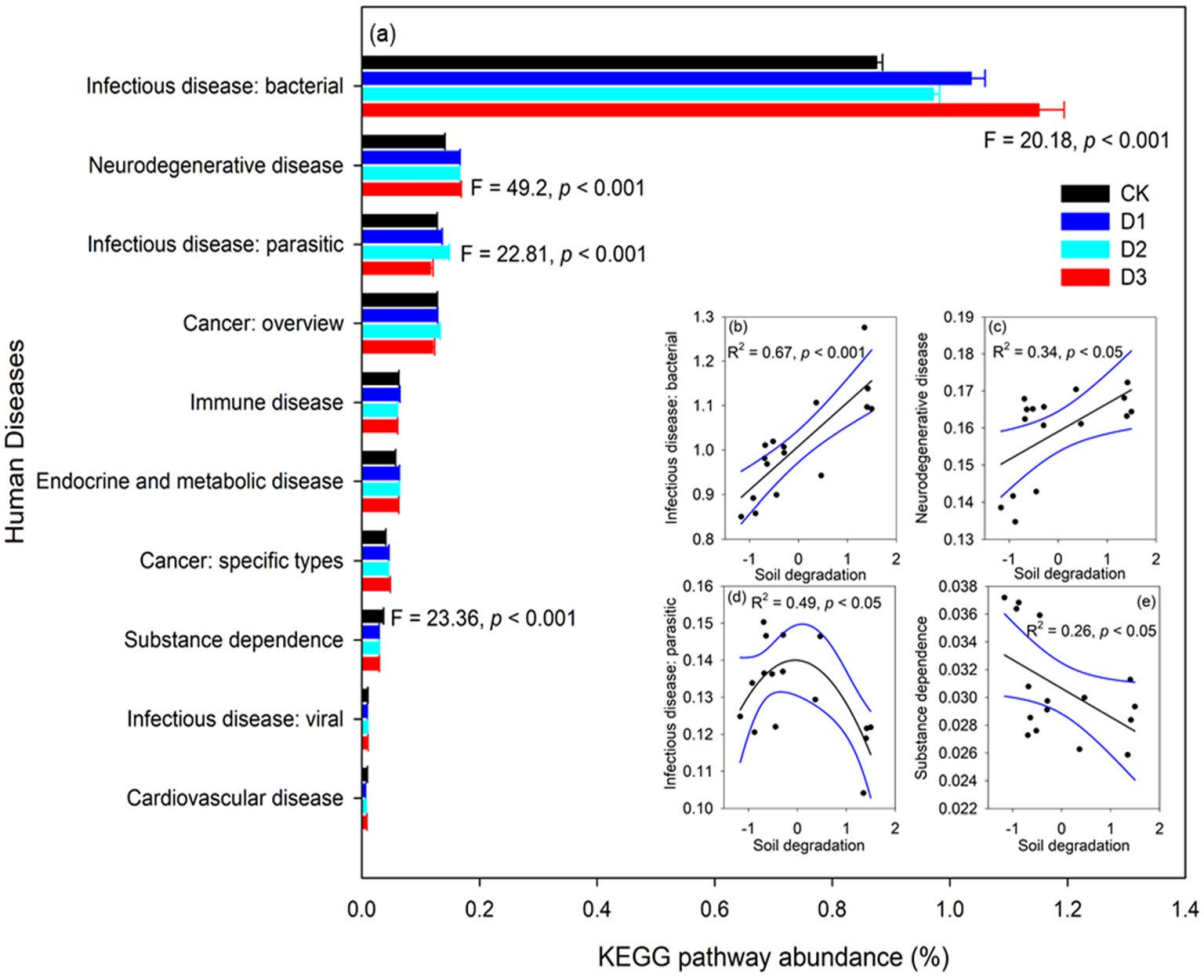
The predicted KEGG category abundance (**a**) of non-degraded (CK), lightly degraded (D1), moderately degraded (D2), and highly degraded (D3) alpine meadows, and the correlation analysis between soil degradation and KEGG categories (**b**–**e**).

We found 10 bacteria related to human diseases (Fig. 7a), and infectious disease: bacterial (F = 20.18, *p* < 0.001) and neurodegenerative disease (F = 49.2, *p* < 0.001) were significantly increased from CK to D3. The metabolic pathway of substance dependence was significantly decreased from CK to D3 (F = 23.36, *p* < 0.001). Infectious disease: parasitic was highest in D2 (F = 22.81, *p* < 0.001). Correlation analysis also showed that infectious disease: bacterial (Fig. 7b; R^2^ = 0.67, *p* < 0.01) and neurodegenerative disease (Fig. 7c; R^2^ = 0.34, *p* < 0.05) were significantly positively correlated with soil degradation. However, infectious disease: parasitic showed a negative quadratic relation with soil degradation (Fig. 7d; R^2^ = 0.49, *p* < 0.05), and the metabolic pathway of substance dependence was significantly negatively correlated with soil degradation (Fig. 7e; R^2^ = 0.26, *p* < 0.05).

## Discussion

### Response of bacterial and fungal properties to different degrees of degradation

Bacterial community structures change significantly as alpine meadow degradation increases due to differences in soil properties and vegetation characteristics among different degraded grasslands^6,15^. With an increase in degradation, the vegetation coverage has been reduced, and soil nutrient contents appear to exhibit a decreasing trend in the Qinghai-Tibetan Plateau^21^. Similarly, the belowground vegetation characteristics (root biomass, root C, root N, and root C/N ratios) and soil properties (soil pH, electrical conductivity, bulk density, soil water content, soil total C, soil total N, and soil total C/N ratios) differed significantly in this study, which corroborates previous studies^24,25^. A decrease in aboveground plant biomass can reduce the litter input to the soil, and restrict the activity and abundance of the soil microbial community^21,26^. We also found that the soil bacterial Shannon diversity decreased greatly in the highly degraded plots. The decreased vegetation biomass due to grassland degradation is likely responsible for decreased C and N concentrations in the soil^27^, which, in turn, decreased the soil bacterial Shannon diversity under the highly degraded plots caused by overgrazing. This was in agreement with previous results obtained by Zhang et al^28^. In addition, the soil bacterial richness appeared to be resistant to soil degradation in this alpine meadow; however, fungal richness decreased significantly in the highly degraded plots compared to the non-degraded plots, implying that the fungal community was more sensitive to degradation than the bacterial community^6^. These differences are attributed to the higher sensitivity of fungi to soil nutrient limitation^29^. Degradation significantly reduced the soil organic carbon content^30^, soil water content^31^, and soil nitrogen content in the present study. Similar to bacterial and fungal studies in QTP grasslands^15^, *Actinobacteria* and *Ascomycota* were the most abundant bacterial and fungal phyla, respectively, detected in these meadows. In addition, the other abundant phyla included *Proteobacteria*, *Acidobacteria* and *Chloroflexi*. *Actinobacteria* species have strong metabolic capacity at low temperatures^32^, and *Acidobacteria* play important roles in organic matter decomposition and nutrient cycles^33,34^. In this study, *Actinobacteria* and *Acidobacteria* were changed significantly in degraded alpine meadows, and this might affect the decomposition and metabolism of soil organic matter. However, no significant differences were found for *Proteobacteria*, which play a key role in ecosystems such as in the oxidation of organic and inorganic compounds^35^. The genus *Mortierella* from the family of *Mortierellaceae* includes fast-growing fungi capable of rapidly invading new substrates^36^ and are often the pioneer colonizers of freshly fallen litter^37^. In this study, plant aboveground biomass decreased significantly from CK to D3, which in turn decreased the litter quantity and the litter decomposition fungi, such as *Mortierella*.

### Relationships between the shifts in soil properties and microbial diversity and richness

The composition of soil microbes can change with plant type changes due to the influence of vegetation growth on soil properties and the alteration of microclimates affecting microbial environments^38,39^. In terrestrial regions, pH is the most vital factor affecting the microbial community and diversity in soils^40,41^. In the present study, the Monte Carlo permutation test suggested that plant root C, soil pH, EC, and soil TC significantly influenced the bacterial communities, while only root biomass and soil TN significantly influenced the fungal communities. In addition, soil pH increased significantly in degraded alpine meadows. Thus, the development of microbial communities could be limited by the high pH caused by grassland degradation^27^. As previously reported^42,43^, *Bacteroidetes* was significantly positively correlated with pH, and this was confirmed in the present study. This result showed that *Bacteroidetes* soil bacteria were highly resistant to land degradation because of their positive correlation with soil pH. Interestingly, root biomass decreased significantly as the degradation level increased. A decrease in root biomass can limit the litter input to the soil, thus restricting the abundance of *Basidiomycota* and *Mucoromycota* under land degradation^37^.

One goal of this research was to evaluate the relationships between soil degradation, plant root characteristics, and bacterial and fungal diversity. Changes in plant diversity and soil properties due to alpine meadow degradation can result in variations in the bacterial community structure and composition^15^. Although soil degradation decreased the bacterial diversity, there was no significant correlation between soil degradation and soil fungal diversity in the SEM, implying that fungal diversity is less influenced by degradation than bacterial diversity. Plant root biomass was significantly negatively and positively correlated with soil bacterial and fungal richness, respectively. Interestingly, plant root C/N ratios showed consistent negative relationships with both soil bacterial and soil fungal richness.

### Relationships between soil degradation and metabolic pathways related to human diseases

As the degradation degree increases, microbes might produce specialized proteins to survive in the degraded alpine steppe environment^15^. Without metagenomic data, the functional capacities of microorganisms cannot be effectively analyzed. Recently, Aßhauer et al.^23^ suggested that Tax4Fun analyses are useful in supplementing 16S rRNA analyses as an inexpensive alternative to PICRUSt analyses. The potential KEGG Orthologue (KO) functional profiles of microbial communities have also been predicted by 16S rRNA gene sequences using PICRUSt^44^. A recent study based on Tax4Fun analyses showed that most bacteria involved in human diseases increase as alpine steppe degradation increases^15^. In this work, we found that infectious disease: bacterial and neurodegenerative disease were significantly positively correlated with soil degradation. Alpine meadows and steppes are the two main livestock grazing regions in the QTP, and overgrazing has caused severe environmental degradation in this area, causing a loss of soil nutrient and plant biodiversity. Infections, including by *Salmonella*, are reportedly suffered by approximately 1.4 million people in the United States as a result of the consumption of contaminated meat^45^. People who live in degraded meadows in the QTP could suffer negative health effects when using livestock without prior examination and treatment^15^, and thus human health might be impacted by livestock grazing on degraded soils.

## Conclusions

This study demonstrates that the plant and soil properties, and the rhizosphere microbiomes under field conditions varied with degradation intensity. The soil pH, EC, and BD values significantly increased as the degradation level increased. The abundances of *Bacteroidetes* and *Gemmatimonadetes* were significantly positively correlated with soil EC, pH, root C/N, and root C, whereas *Basidiomycota* and *Mucoromycota* were significantly negatively correlated with soil pH and EC. Soil degradation caused significant shifts in bacterial diversity and fungal richness, but did not significantly alter bacterial richness and fungal diversity in alpine meadow of the QTP. Plant litter mass and root C/N ratio proved to be the important factors controlling the shifts in bacterial and fungal diversity and richness. This study provides an improved understanding of the adapatation of the microbial community to soil degradation caused by overgrazing.

## Materials and methods

### Study area

**Figure 8:**
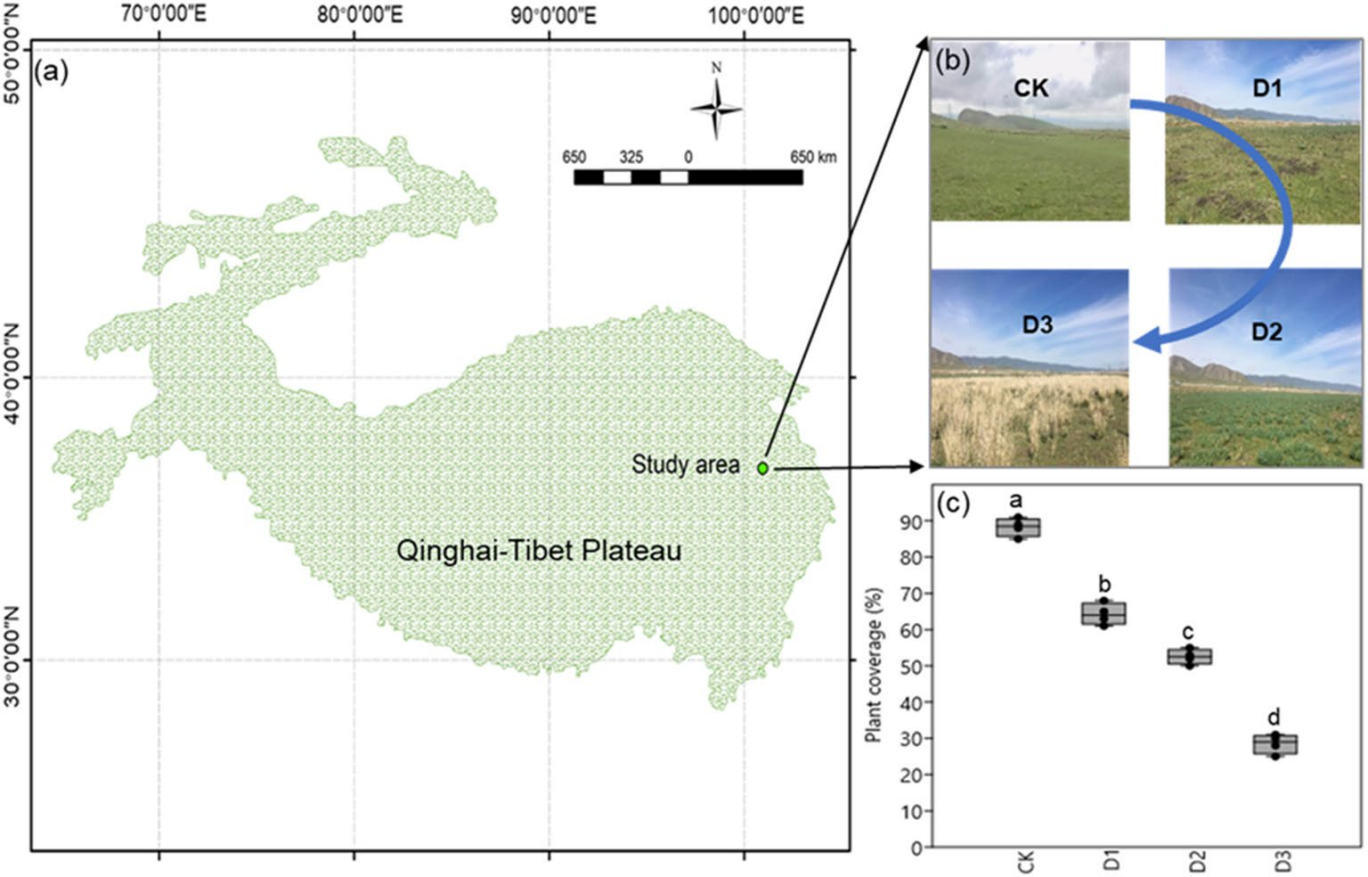
Schematic of the field experimental designs in the study area (**a**), and the distance from the watering point (**b**) representing the degradation intensity (lightly degraded, D1; moderately degraded, D2; and highly degraded, D3), and the plant total coverage (**c**). The photographs were taken by Chao Yang at Haibei prefecture (36°55′N, 100°57′E), and the map was created by Chao Yang with ArcGIS 10.2 for Desktop (http://www.esri.com/arcgis/about-arcgis).

The field experiment was conducted in the Haibei prefecture (36° 55′ N, 100° 57′ E, 3040 m el.), Qinghai Province, northeast of Tibetan Plateau (Fig. 8a). The mean annual temperature and mean annual rainfall at this site are − 0.45 °C and 400 mm, respectively. This area is dominated by meadow vegetation, including *Stipa capillata*, *Poa pratensis,* and *Kobresia* spp. The study site was grazed by 20 yaks (*Bos grunniens*) in a year-long continuous grazing system (5 ha), and the grazing intensity was four yaks ha^–1^ year ^–1^, which is a high grazing intensity according to Wang, et al.^46^. We selected four sites with different degradation levels to represent the degradation intensity (Fig. 8b): non-degraded (CK), lightly degraded (D1), moderately degraded (D2), and highly degraded (D3). The plant total coverage was 88.25% (CK), 64.25% (D1), 52.5% (D2), and 28.5% (D3) (Fig. 8c). Details of the soil physiochemical properties are presented in Table S1.

### Plant root biomass and soil sampling

The field investigation was conducted in late-July 2019, which is the period of maximum annual biomass. Across each of the four degradation intensities (CK, D1, D2, and D3), four replicate points were separated at intervals of 50 m, and the sampling points from D1 to D3 were also separated at intervals of 50 m. In each point, we set up three random quadrats (1 m × 1 m) to measure the vegetation coverage and obtain root and rhizosphere soil samples. In total, there were 16 plots (four degradation intensities × four replicates) and 48 quadrats.

Plant total coverage was calculated by the ratio of the physically shaded area of the observed species in each quadrat to the total area of a quadrat^47^. The aboveground litter mass (LM) was collected and weighed. The belowground root biomass at 0–20 cm depth was obtained using a 10-cm-diameter root auger in each quadrat after measurement of plant coverage. Additionally, in each quadrat, a mixed rhizosphere soil sample at 0–20 cm depth from three random samples was collected using a 3-cm-diameter soil auger. The constant weight of the aboveground biomass and root biomass (RB) was obtained by oven-drying at 65 °C for 24 h after removal of the attached soil.

### Plant root and soil properties analyses

Root carbon (RC), root nitrogen (RN), soil total carbon (TC), and soil total nitrogen (TN) concentrations were measured using a CHNS Element Analyzer (Elementar, Germany). Soil pH and electrical conductivity (EC) were measured using a glass electrode in a 1:2.5 soil: water suspension. Soil bulk density (BD) was calculated using the ring knife method at 0–20 cm depth. A foil sampler with a volume of 100 cm^−3^ was used to obtain the samples, which were then oven-dried at 105 °C for 24 h. Soil water content (SWC) was measured gravimetrically and expressed as a percentage of soil water to dry soil weight.

### DNA extraction and PCR amplification

The bacterial and fungal extraction and determination methods were similar to those used in a previous study^19^. In brief, the genomic DNA was extracted from each soil sample using a Fast DNA SPIN Kit for soil (MP Biomedicals, CA, USA). We weighed a 0.30 g soil sample from each treatment. Soil DNA integrity was then detected by 0.8% agarose gel electrophoresis. The V3 and V4 regions of the bacterial 16S rRNA gene were amplified using universal primers 338F and 806R, and the fungal ITS gene was amplified by ITS1 primers. The PCR amplification of the 16S rRNA gene and ITS1 region included pre-denaturation at 95 °C for 3 min; 27 cycles at 95 °C for 30 s, annealing at 55 °C for 30 s, elongation at 72 °C for 45 s, and an extension at 72 °C for 10 min.

### Processing of MiSeq sequencing data

Purified amplicons were pooled in equimolar ratios and paired-end sequenced on an Illumina MiSeq PE300 platform(Illumina, San Diego,USA) according to the standard protocols by Majorbio Bio-Pharm Technology Co. Ltd. (Shanghai, China). Processing of the raw data and diversity indices including Shannon and Chao1 richness were implemented in QIIME (ver. 1.3.0)^48^. The reads were truncated to obtain an average quality score lower than 20; a sliding window over 50 bp and over 3 continuous bases, and the reads with lengths fewer than 300 bp were discarded as well. We then used UPARSE (ver. 7.1) to cluster the high-quality sequences with a 97% identity threshold into operational taxonomic units (OTUs)^49^. The original sequencing data of the bacteria and fungi were deposited into the NCBI Sequence Read Archive (SRA) database (Accession Number: SRP251789).

### Statistical analysis

Significance analyses of litter mass, plant root indices (RB, RC, RN, and RC/N), soil indices (pH, EC, BD, SWC, SOC, TN, C/N, and soil degradation), and microbial diversity indices (Shannon and Chao 1) between four degradation levels were performed using one-way analysis of variance (ANOVA) in PAST (ver. 3.25; Natural History Museum, University of Oslo, Norway). The level of significance was tested at *p* < 0.05 using Tukey’s pairwise test.

Since soil indices within each degradation are often closely correlated, we conducted principal component analyses (PCA) to create multivariate indexes for each degradation level, reducing collinearity among the predictor variables^50^. Therefore, we used the eigenvalues of PCA axis 1 to characterize soil degradation (Figure S2). Additionally, the correlation between the soil degradation and plant root biomass and root C/N ratios was analyzed using an exponential model in PAST (ver. 3.25).

Nonmetric multidimensional scaling (NMDS) based on Bray–Curtis similarity matrices was performed to identify the total structural changes in soil bacteria and fungi, and significance was tested by a permutational multivariate ANOVA (PERMANOVA) in PAST (ver. 3.25).

The relationship between litter and root properties (LM, RB, RC, RN, and RC/N), soil properties (pH, EC, BD, SWC, and C/N) and soil microbial community (bacteria and fungi) was determined by redundancy analysis (RDA) using CANOCO (ver. 4.5, Plant Research International, Wageningen, Netherlands). The significance of the effect of each variable was defined using Monte Carlo permutation tests (permutations = 999), and the resulting significance level was tested by the F- and *p*-values. The causal relationships among soil degradation, plant root indices, and soil microbial diversities and richness were quantified by structural equation modeling (SEM) using AMOS (ver. 21.0, IBM, SPSS, Inc., Chicago, IL, USA). A non-significant chi-square (χ^2^) test and the root mean square error of approximation (RMSEA) were used to define the goodness of fit of the model according to Yang et al.^19^. We used 16S rRNA gene sequences to calculate the metabolic pathways related to human diseases in the Kyoto Encyclopedia of Genes and Genomes database^51^ (KEGG, www.kegg.jp/kegg/kegg1.html) using the Tax4fun package in R (ver. 3.5.1). Additionally, the correlation between the soil degradation and the metabolic pathways related to human diseases was analyzed using a linear model in PAST (ver. 3.25).

## Supplementary Information

Supplementary Information.
