# Supplementary material for: Soil degradation influences soil bacterial and fungal community diversity in overgrazed alpine meadows of the Qinghai-Tibet Plateau
Source: Sci Rep. 2021 Jun 2;11:11538. doi: 10.1038/s41598-021-91182-7 (PMC8172827; doi:10.1038/s41598-021-91182-7)
Supplement: Supplementary file 1 — Supplementary Information. [file 41598_2021_91182_MOESM1_ESM.docx]

**Soil degradation influences soil bacterial and fungal community** **diversity in overgrazed alpine meadows of the Qinghai-Tibet Plateau**

**Lin Dong** ^a,1^**, Jingjing Li** ^a,1^**,** **Juan Sun**^1^**, Chao Yang**^1,^**^*^**

^1^ Grassland Agri-Husbandry Research Center, College of Grassland Science, Qingdao Agricultural University, Qingdao 266109, China

^a^ These authors contributed equally to this work

* Correspondence: yangchao@qau.edu.cn (Chao Yang)

Supplementary Material

# Supplementary Figures and Tables


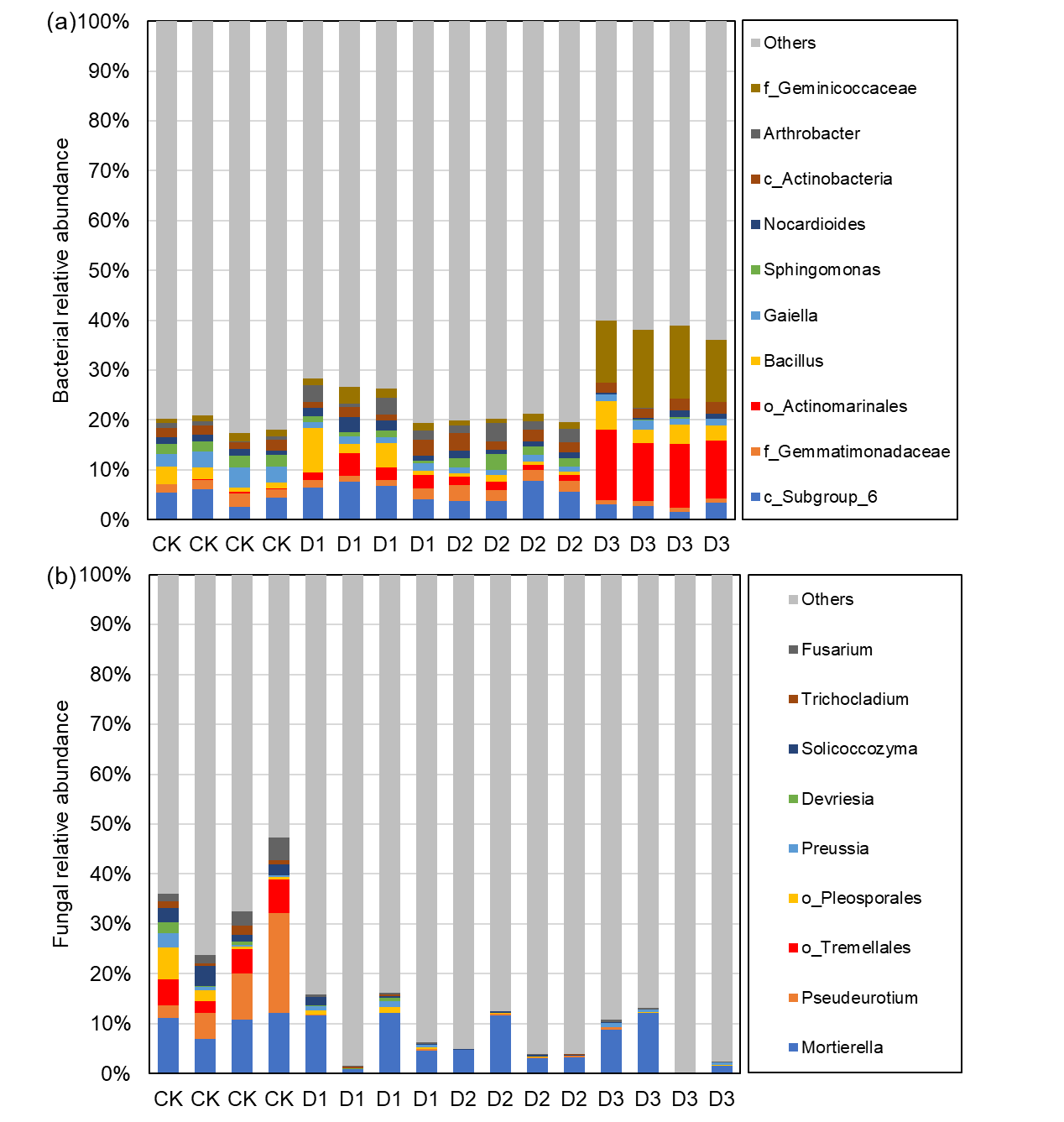


**Fig. S1** Relative abundances of the soil bacterial (a) and fungal (b) genera in non-degraded (CK), lightly degraded (D1), moderately degraded (D2), and highly degraded (D3) alpine meadows. Due to the limitation of classification level, some genera of microorganisms have not been accurately identified, so they can only be expressed to the corresponding class, order or family level.


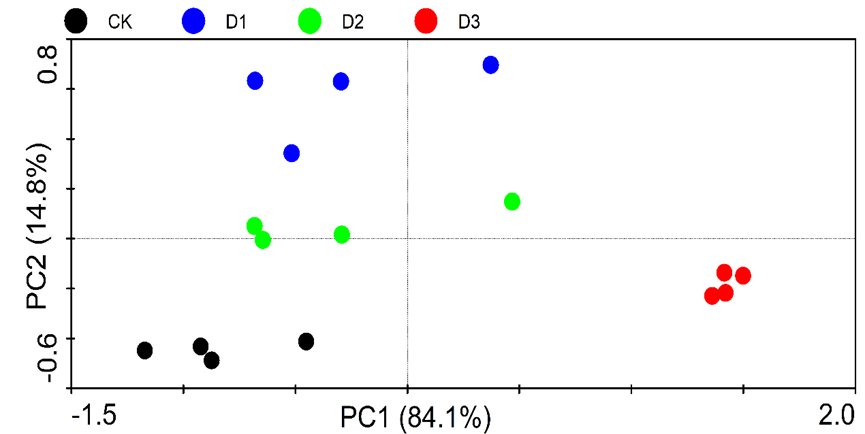


**Fig. S2** The principal component analyses (PCA) was used to create multivariate indexes for each degradation level, and the eigenvalues of PCA axis 1 was used to characterize the soil degradation.

**Table S1.** One-way ANOVA of the soil properties of non-degraded (CK), lightly degraded (D1), moderately degraded (D2), and highly degraded (D3) site. Values are mean ± standard error.

|  | pH | EC (μs cm^-1^) | BD (g cm^-3^) | SWC (%) | TC (g kg^-1^) | TN (g kg^-1^) | C/N ratio |
| --- | --- | --- | --- | --- | --- | --- | --- |
| CK | 7.85 (0.03) | 274 (52) | 0.99 (0.02) | 20.60 (0.36) | 38.62 (0.56) | 3.75 (0.04) | 10.30 (0.18) |
| D1 | 8.16 (0.17) | 671 (216) | 1.04 (0.02) | 18.35 (0.21) | 79.12 (7.3) | 3.04 (0.29) | 26.15 (1.37) |
| D2 | 8.14 (0.03) | 642 (238) | 1.10 (0.02) | 17.90 (0.23) | 53.42 (0.59) | 3.26 (0.04) | 15.65 (0.48) |
| D3 | 8.73 (0.01) | 3901 (149) | 1.22 (0.01) | 15.58 (0.23) | 33.74 (1.31) | 2.99 (0.11) | 11.30 (0.11) |
| F-value | 17.01 | 89.12 | 36.92 | 61.02 | 29.54 | 4.748 | 97.47 |
| *p-*value | ** | ** | ** | ** | ** | * | ** |

Note: EC (electrical conductivity), BD (bulk density), SWC (soil water content), TC (total carbon), TN (total nitrogen). In the list, the significant relationships at *p* < 0.05 and < 0.01 were indicated by * and ** using the Tukey’s pairwise test.

**Table S2** The Monte Carlo permutation tests (permutations = 499) among plant root, soil the properties and soil microbial communities. The significance level was tested by the F- and p-values.

|  | Bacterial community | |  | Fungal community | |
| --- | --- | --- | --- | --- | --- |
|  | F-ratio | p-value |  | F-ratio | p-value |
| LM | 0.41 | 0.746 |  | 0.17 | 0.889 |
| RB | 1.56 | 0.19 |  | **4.89** | **0.012** |
| RC | **8.9** | **0.001** |  | 0.64 | 0.551 |
| RN | 1.12 | 0.337 |  | 0.83 | 0.455 |
| RC/N | 0.63 | 0.576 |  | 1.96 | 0.157 |
| pH | **3.09** | **0.03** |  | 1.13 | 0.344 |
| EC | **3.25** | **0.022** |  | 0.46 | 0.628 |
| BD | 0.21 | 0.858 |  | 2.44 | 0.089 |
| SWC | 1.95 | 0.11 |  | 0.82 | 0.457 |
| TC | **5.11** | **0.014** |  | 0.76 | 0.487 |
| TN | 1.14 | 0.345 |  | **5.39** | **0.009** |
| C/N | 1.01 | 0.41 |  | 0.25 | 0.779 |
